# METTL16-mediated translation of *CIDEA* promotes non-alcoholic fatty liver disease progression *via* m6A-dependent manner

**DOI:** 10.7717/peerj.14379

**Published:** 2022-12-01

**Authors:** Jinhong Tang, Xiangyun Zhao, Wei Wei, Weiwei Liu, Huining Fan, Xiu ping Liu, Yungai Li, Long Wang, Jinghui Guo

**Affiliations:** 1 Department of Gastroenterology, Shanghai Sixth People’s Hospital Affiliated to Shanghai Jiao Tong University School of Medicine, Shanghai, P.R. China; 2 Current Affiliation: Endoscopy Center, Minhang Hospital, Fudan University, Shanghai, P.R. China; 3 Digestive Endoscopic Center, Shanghai Sixth People’s Hospital Affiliated to Shanghai Jiao Tong University School of Medicine, Shanghai, P.R. China; 4 Department of Gastroenterology, Shanghai Fudan University Affiliated Fifth People’s Hospital, Shanghai, China; 5 Department of Clinical Laboratory, Shanghai Sixth People’s Hospital Affiliated to Shanghai Jiao Tong University School of Medicine, Shanghai, P.R. China

**Keywords:** Non-alcoholic fatty liver disease, N6-methyladenosine (m6A), METTL16, CIDEA, m6A sequencing

## Abstract

**Background:**

As the most prevalent chemical modifications on eukaryotic mRNAs, N6-methyladenosine (m6A) methylation was reported to participate in the regulation of various metabolic diseases. This study aimed to investigate the roles of m6A methylation and methyltransferase-like16 (METTL16) in non-alcoholic fatty liver disease (NAFLD).

**Methods:**

In this study, we used a model of diet-induced NAFLD, maintaining six male C57BL/6J mice on high-fat diet (HFD) to generate hepatic steatosis. The high-throughput sequencing and RNA sequencing were performed to identify the m6A methylation patterns and differentially expressed mRNAs in HFD mice livers. Furthermore, we detected the expression levels of m6A modify enzymes by qRT-PCR in liver tissues, and further investigated the potential role of METTL16 in NAFLD through constructing overexpression and a knockdown model of METTL16 in HepG2 cells.

**Results:**

In total, we confirmed 15,999 m6A recurrent peaks in HFD mice and 12,322 in the control. Genes with differentially methylated m6A peaks were significantly associated with the dysregulated glucolipid metabolism and aggravated hepatic inflammatory response. In addition, we identified five genes (*CIDEA*, *THRSP*, *OSBPL3*, *GDF15* and *LGALS1*) that played important roles in NAFLD progression after analyzing the differentially expressed genes containing differentially methylated m6A peaks. Intriguingly, we found that the expression levels of METTL16 were substantially increased in the NAFLD model *in vivo* and *in vitro*, and further confirmed that METTL16 upregulated the expression level of lipogenic genes *CIDEA* in HepG2 cells.

**Conclusions:**

These results indicate the critical roles of m6A methylation and METTL16 in HFD-induced mice and cell NAFLD models, which broaden people’s perspectives on potential m6A-related treatments and biomarkers for NAFLD.

## Introduction

Non-alcoholic fatty liver disease (NAFLD) has become the most common cause of chronic liver disease worldwide, affecting 20–30% of the general population and up to 75% in those with obesity (Younossi et al., 2018). NAFLD encompasses a spectrum of pathological changes, from simple steatosis to non-alcoholic steatohepatitis (NASH), subsequently progressing to fibrosis and cirrhosis, and up to 27% in those with cirrhosis will develop hepatocellular carcinoma (HCC) (Raza et al., 2021). Given the high prevalence of NAFLD, it is predicted that NAFLD could become the most primary indication for liver transplantation by 2030 (Oliveros-Montiel, Santos-López & Sedeño-Monge, 2020). However, apart from controlling the risk factors such as weight loss, diet modification, and probably applying lipid-lowering drugs and insulin sensitizers, there are currently no effective mechanistic treatments for NAFLD (Raza et al., 2021).

N6-methyladenosine (m6A) methylation denotes the addition of a methyl group on the N6 site of adenosine within RNA, which is the most prevalent chemical modification of eukaryotic mRNAs that can be observed in multitude species, including viruses, bacteria, mammals and humans (Wang, Sha & Sun, 2021). Based on the development of Methylated RNA Immunoprecipitation sequencing (MeRIP-seq) (Meyer et al., 2012), accumulating studies have demonstrated that m6A methylation can modulate a variety of RNA metabolisms, including RNA transport (Wen et al., 2018), splicing (Bartosovic et al., 2017), stability (Genenncher et al., 2018), translation (Liu et al., 2020) and microRNA processing (Chen et al., 2020). Ultimately, post-transcriptional RNA modification can further influence cellular physiology and various biological progresses, including embryonic stem cell differentiation, immunomodulation and tumorigenesis (Wang, Sha & Sun, 2021; Wen et al., 2018; Liu et al., 2020; Geula et al., 2015; Zheng et al., 2017). These dynamic and reversible modifications are catalyzed by the methyltransferase complex called “writers” consist of METTL3 (methyltransferase-like 3) (Peng et al., 2021), METTL14 (methyltransferase-like 14) (Chen et al., 2020), METTL16 (methyltransferase-like 16) (Pendleton et al., 2017), WTAP (Wilms tumor 1 associated protein) (Chen et al., 2019), and are removed by the demethylases complex called “erasers” composed of ALKBH5 (alkB homolog5) and FTO (fat mass and obesity-associated protein). Additionally, m6A exerts many functions through m6A-binding proteins called “readers”, among which the best described are the YTH domain family proteins YTHDF1/2/3 (Liu et al., 2020) and HNRNP (heterogeneous nuclear ribonucleoprotein C) (Peng et al., 2019). Notably, recent evidence has indicated that m6A methylation and its modulators play important roles in various metabolic diseases *via* regulating glucolipid metabolism and inflammatory processes (Peng et al., 2019; Luo et al., 2019; Yang et al., 2019; Li et al., 2020; Zhang et al., 2020a; Zhong et al., 2018). For example, FTO promoted the expressions of lipogenic genes by erasing their m6A modification in patients with type 2 diabetes (T2DM) (Yang et al., 2019). Zhong et al. (2018) revealed that m6A methylation affected circadian regulation of lipid metabolism by mediating mRNA lifetime and expression of the nuclear receptor peroxisome proliferator-activator-α (PPaR-α) in murine liver. Therefore, further investigation is warranted to determine the effects of m6A methylation modification and its modify enzymes in the development of NAFLD.

**Figure 1:**
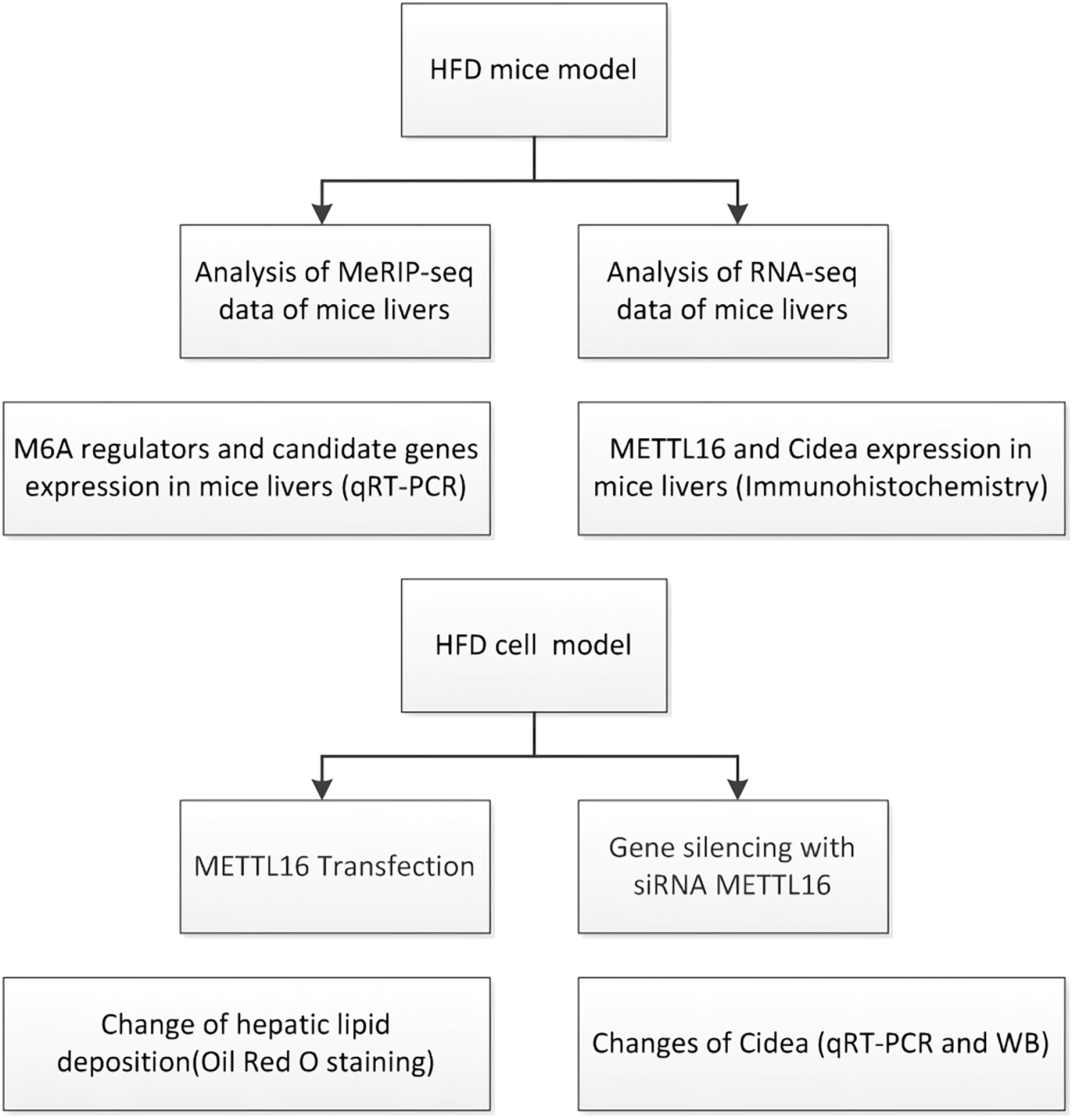
Flowchart showing the experimental procedure.

In this study (Fig. 1), we carried out the MeRIP-seq analysis to illustrate the m6A methylation patterns in HFD-induced NAFLD mice models. We also explored the underlying mechanism between m6A modification and NAFLD through identifying the downstream target genes and signaling pathways. Furthermore, we evaluated the expression levels of major m6A regulators by qRT-PCR analysis to explore the potential driving forces behind m6A methylation. Finally, we validated the vital roles of METTL16 in the progress of NAFLD through constructing overexpression and knockdown of METTL16 model in HepG2 cells.

## Materials and Methods

### Animal experiments

Six 6-week-old male C57BL/6J mic were used in this study. These mice were provided by Shanghai Slack Laboratory Animal LTD and housed at 21–23 °C with a 12 h light/12 h dark cycle and 55% ± 5% humidity in the general animal laboratory of Shanghai Jiao Tong University Affiliated Sixth People’s Hospital. The animal protocol was approved by the Animal Care and Ethics Committee of Shanghai Jiao Tong University Affiliated Sixth People’s Hospital (IACUC 2020-0035). These mice were randomly divided into two groups as determined by the principle of minimum sample size (*n* = 3). Three mice fed with standard low-fat diet (D12450B, 10% kcal fat/35% sucrose; Research Diets Inc., New Brunswick, NJ, USA) were considered as the control (*n* = 3). Another three mice fed with high-fat diet (D12492, 60% kcal fat; Research Diets Inc., New Brunswick, NJ, USA) (Kennedy et al., 2018) were used as the experimental group (*n* = 3) to establish NAFLD mice model. The weight and fast glucose of each mouse were measured on the day of initiation and 12 weeks after intervention. These experimental mice were provided with a comfortable living environment and adequate food and water to minimize their discomfort and pain. After 12 weeks of feeding, we euthanized these mice to ensure compliance with animal ethics: we killed these mice by cervical dislocation after intraperitoneal injection of chloral hydrate. The liver tissues were separated and immediately frozen in liquid nitrogen and kept at −80 °C before use.

### H&E staining

The liver tissues were fixed with 4% paraformaldehyde, embedded in paraffin wax, and then cut into 8 $\mu m$ sections. These slices were next stained with hematoxylin and eosin (H&E) and observed under a microscope to investigate the histological changes in HFD mice livers.

### Oil red O staining

The liver tissues were embedded in optimum cutting temperature compound (Tissue-Tekcryomold, CA, USA) and rapidly frozen cut into 5 $\mu m$ sections. These slices were stained with Oil Red O solution for 10 min as previous description (Luo et al., 2019) and then observed under a microscope. Finally, we use the ImageJ software to quantify the outcome of the hepatic lipid accumulation in HFD mice.

### RNA extraction and RNA-seq

Total RNA was extracted from liver tissues using TRIzol reagent (Invitrogen Life Technologies, Carlsbad, CA, USA) and then precipitated with isopropanol and ethanol. NanoDrop ND-2000 (Thermo Fisher Scientific, Waltham, MA, USA) was used to assess the quantity and quality of total RNA. The mRNA was isolated from total RNA samples by using the Seq-Star T Mpoly(A) mRNA Isolation Kit (Arraystar, Rockville, MD, USA), followed by 50 bp single-end sequencing under the BGISEQ-500 platform. The differentially expressed genes (DEGs) were further conformed by RNA-seq analysis. The statistical power of this experimental design, calculated in RNASeqPower is 0.848676.

### qRT-PCR analysis

Total RNA was reverse transcribed into cDNA using a cDNA Synthesis Kit (TransGen Biotech, Beijing, China) according to the manufacturer’s instructions. The resulting cDNA was used for reverse transcription quantitative real-time PCR (qRT-PCR) analysis by using the SYBR premix Ex Taq (Takara, Dalian, China). The mRNA levels were normalized to Glyceraldehyde 3-phosphate dehydrogenase (GAPDH) and quantified using the 2^−ΔΔCt^ method (Yue et al., 2016). All primers used for the amplification are listed in Supplemental Table S1.

### MeRIP-seq and data analysis

MeRIP-seq, developed in 2012 by Meyer KD, combines RNA-protein co-immunoprecipitation with high-throughput sequencing technology to achieve RNA methylation analysis at the whole transcriptome level, which was favorable for further revealing the potential biological functions of m6A modification in a new research area (Meyer et al., 2012). MeRIP-seq analysis was provided by Shanghai Cloudseq Biotech Inc. Briefly, fragmented mRNA was immunoprecipitated using the GenSeq™ m6A RNA IP Kit (GenSeq Inc., Shanghai, China). Then, input samples and m6A immunoprecipitated RNA samples were used to build up the RNA sequencing library following manufacturer’s instructions of NEBNext® Ultra II Directional RNA Library Prep Kit (New England Biolabs Inc., Ipswich, MA, USA). Next, libraries were sequenced on an Illumina Hiseq 4000 platform with paired-end 150 bp read length. Finally, the length and methylated sites on RNA were identified as previously described (Luo et al., 2019). The biological function of differential m6A methylation sites were predicted by gene ontology (GO) analysis and pathway enrichment analysis from GO (http://geneontology.org/) and Kyoto Encyclopedia of Genes and Genomes (KEGG) database (www.genome.jp/kegg).

### Immunohistochemistry

Paraffin-embedded liver sections from six mice were used to perform the immunohistochemistry analysis according to the manufacturer’s instructions. After dewaxing, hydration, removing the possible interference of endogenous enzyme and antigen repair, we performed blocking and incubation of antibody on the paraffin sections. To detect the expression of CIDEA, liver sections were incubated with a rabbit anti-CIDEA antibody (O60543; CUSABIO, Wuhan, China) overnight at 4 °C and then incubated with a biotin-labeled secondary antibody. To detect the expression of METTL16, liver sections were incubated with a rabbit anti-METTL16 antibody (O86W50; PROTEINTECH, Chicago, IL, USA) overnight at 4 °C and then incubated with a peroxidase-labeled secondary antibody. The staining density was observed under a microscope and calculated using the ImageJ software. Three fields were analyzed from each sample.

### Cell culture and treatments

Human hepatocellular carcinoma cell lines (HepG2) were provided by Shanghai Asia-Vector Biotechnology LTD and cultured in a humidified atmosphere of 5% CO_2_ at 37 °C using Dulbecco’s modified Eagle’s medium (DMEM, Gibco, Carlsbad, CA, USA) supplemented with 1% penicillin-streptomycin solution and 10% fetal bovine serum (FBS). To induce fat-overloading models in HepG2 cells, a BSA-conjugated mixture of free fatty acid (FFAs) was uesed in the medium: 20 mmol/L palmitate acid (PA) and 10 mmol/L oleic acid (OA) stock solution were mixed as a 15 mmol/L FFAs solution (Chung et al., 2019). After treated with FFAs solution for 24 h, cells were washed twice with phosphate-buffered saline (PBS), fixed with 4% paraformaldehyde, washed with 60% isopropanol and then subjected to Oil Red O staining for 10 min.

### METTL16 transfection

For *in vitro* studies, METTL16 overexpression was achieved by transfection with pcDNA3.1 (Asia-Vector Biotechnology LTD, Shanghai, China) using lipo2000 Transfection (Thermo Fisher Scientific, Waltham, MA, USA) reagents according to the manufacturer’s protocol. The primer sequences used for METTL16 amplification were GGTGAATTCCTCGAGGCCACCATGGCTCTGAGTAAAT (forward) and AGAGGGGCGGGATCCCTAGTTAACTGCAACAAGCCT (reverse). After incubation for 8 h, lipid containing plasmid was removed and warmed complete medium was added. Cells were transfected by 2 $\mu g$ plasmid for another 24 h following transfection. Cells were starved overnight and treated with BSA/FFAs for 24 h before analysis.

### Gene silencing with METTL16 siRNA

siRNA were purchased from Asia-Vector Biotechnology (Shanghai, China). METTL16 siRNA was designed and synthesized as follows: 5′-TGATGGATGCTCTTAAAGA-3′. Cell lines were transiently transfected 20 nM METTL16 siRNA and non-silencing siRNA by using Lipofectamine2000 reagent. Cells were then treated as previously mentioned.

### Western blot

HepG2 cells were washed using PBS and dissolved in lysis buffer supplemented with 50 mmol/L Tris-HCl, 150 mmol/L NaCl and 0.1% sodium dodecyl sulfate (SDS). For Western blot analysis, we used the following primary antibodies: anti-CIDEA antibody (O60543;CUSABIO, Wuhan, China) and anti-METTL16 antibody (O86W50; PROTEINTECH, Chicago, IL, USA). The nuclear and cytoplasmic proteins were separated on a 10% SDS-PAGE and then transferred onto nitrocellulose membranes. The membranes were blocked with 5% skimmed milk for 1 h at room temperature and followed by incubated with primary and secondary antibodies. Finally, we used the Clarity Western ECL substrate (Bio-Rad, Hercules, CA, USA) and Amersham Imager 600 (GE Healthcare, Chicago, IL, USA) to detect the density of the blots.

### Statistical analysis

All the data in our study were expressed as the standard error of the mean (SEM) and statistically analyzed using GraphPad Prism Version 8.0 software. Student’s t-test was conducted to analyze the statistical differences between two groups. Data were considered statistically significant as follows: *P-value < 0.05, **P-value < 0.01 and ***P-value < 0.001.

## Results

### Overview of m6A methylation map within mRNA in HFD mice livers

**Figure 2:**
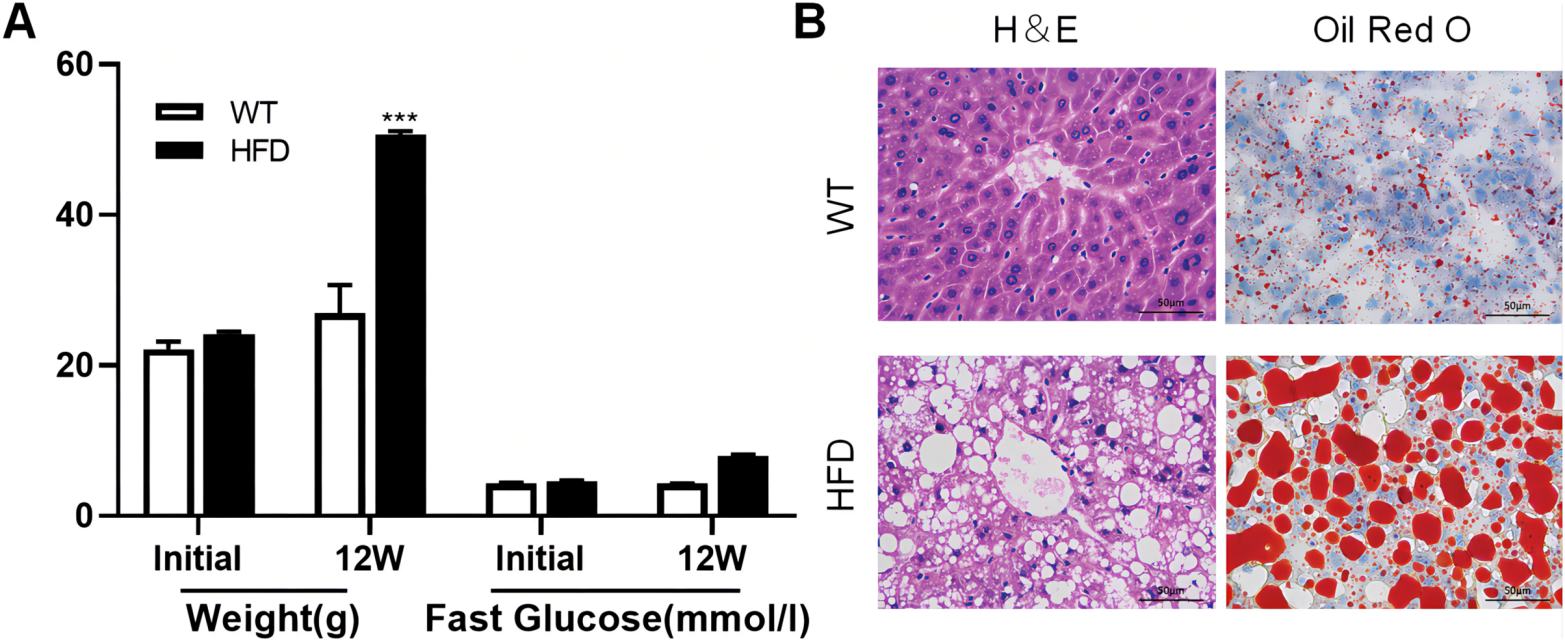
High fat diet (HFD)-induced NAFLD model was successfully established in the liver of mouse. (A) The weight and fast glucose of mice on the day of initiation and 12 week after intervention (*n* = 3). (B) Representative microscopic images of liver sections stained with Oil Red O and H&E (scale bars, 50 $\mu m$). ***P < 0.001. The data in (A) are presented as the means ± SEM. WT, wide type; HFD, high fat diet; H&E, hematoxylin-eosin; NAFLD, Non-alcoholic Fatty Liver.

**Figure 3:**
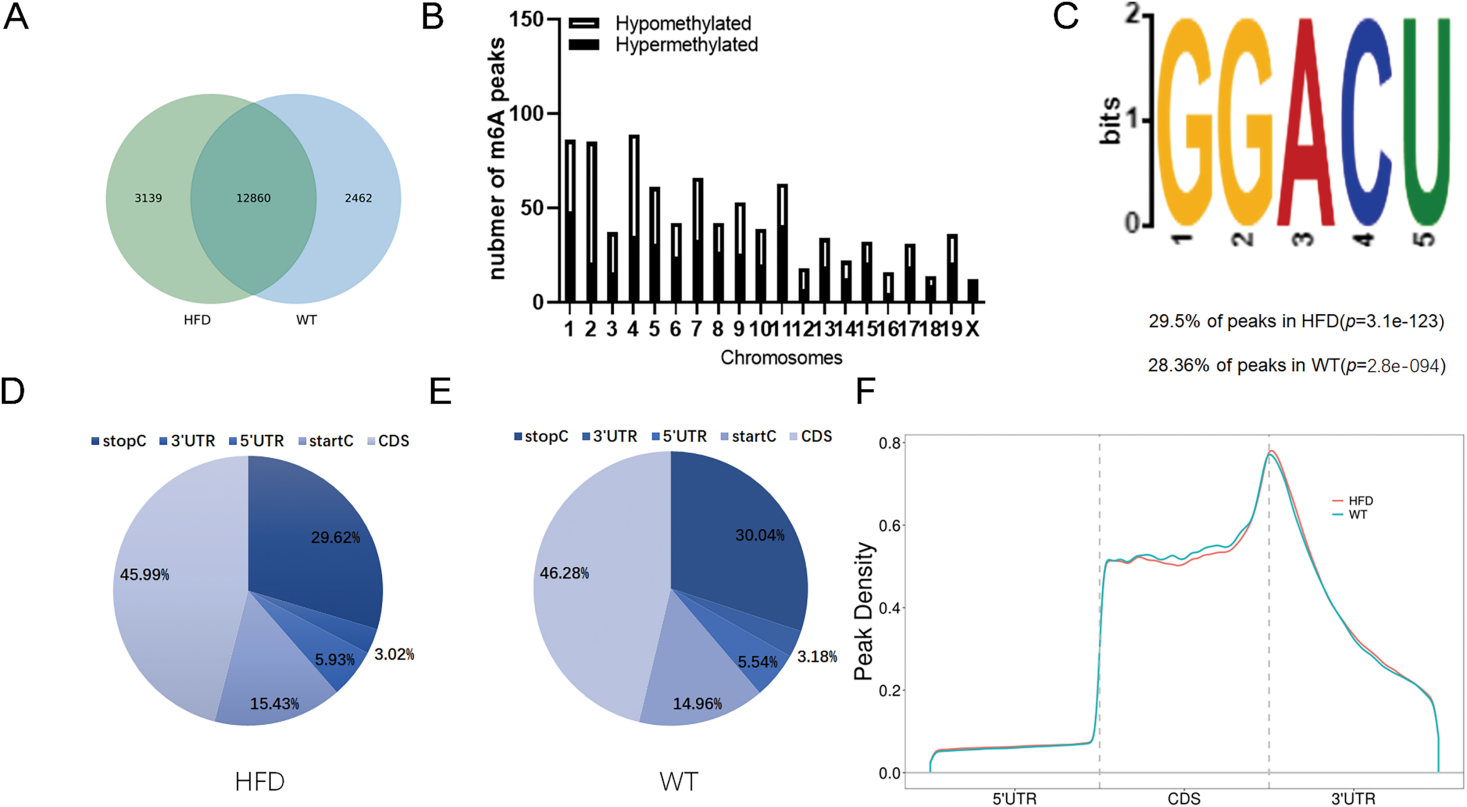
Overview of m6A methylation map within mRNA in HFD mice livers. (A) Venn diagram displaying the distinguished m6A peaks between HFD and WT mice livers. (B) Distributions of hypermethylated and hypomethylated m6A peaks in chromosomes. (C) The most common consensus motif (GGACU) confirmed from the altered m6A peaks of HFD and WT mice livers. (D and E) Pie charts showing the distribution of m6A methylated sites in HFD and WT mice livers. (F) Distributions of the region of average m6A peaks throughout the whole transcriptome in HFD and WT mice livers. CDS, Coding sequence; 3′UTR, 3′untranslated region; 5′UTR, 5′untranslated region.

To illustrate whether the m6A methylation was engaged in NAFLD progression, we first constructed the HFD-induced NAFLD mice model as previously described (Kennedy et al., 2018). Compared to normal feeding, HFD resulted in an approximately 2.5-fold increase in weight (*P* = 0.0004), but no significant increase in fast glucose (Fig. 2A). The microscopic images of liver tissues stained with H&E showed that HFD led to the accumulation of lipid droplets vacuoles in liver tissues (Fig. 2B). Similar observation were observer in liver sections stained with Oil Red O (Fig. 2B), indicated that HFD-induced NAFLD mice model was successfully established. Next, we performed MeRIP-seq analysis of mRNA samples from six mice and identified the repeated m6A peaks as high-confidence m6A methylated sites for subsequent research. Altogether, we confirmed 18,461 m6A peaks including 15,999 m6A peaks in HFD mice and 15,322 peaks in WT mice, with 12,860 peaks overlapped among two groups (Fig. 3A). The distinguished m6A methylated patterns between two groups were displayed in Fig. 3A (2,462 in control and 3,139 in HFD mice). The first 20 differentially methylated m6A peaks are presented in Table 1. Chromosomal distributions analysis revealed that both hypermethylated and hypomethylated m6A peaks were transcribed from all chromosomes, particularly from chr1, chr2 and chr4 (Fig. 3B). To investigate if these peaks contained the classic m6A consensus sequence RRm6ACU (R = A or G), we analyzed the top 5,000 most significant RNA methylation sites. Finally, 1,476 and 1,418 RNA methylation sites were identified on GGACU consensus sequence in HFD and WT mice, respectively (Fig. 3C). Consistent with previous reports (Zheng et al., 2017), it seems necessary for m6A mRNA modification to present the consistent sequence motif. To further detect the preferential distribution of m6A methylated sites throughout the whole transcriptome, these peaks were divided into five transcript fragments, including 5′untranslated region (5′UTR), 3′untranslated region (3′UTR), coding DNA sequence (CDS), start codon segment and stop codon segment. In HFD mice, m6A peaks were enriched within CDS (45.99%), stop codon (29.62%), start codon (15.43%), and followed by 5′UTR (5.93%) and 3′UTR (3.02%) (Fig. 3D). In the control group, m6A peaks were enriched within CDS (46.28%), stop codon (30.04%), start codon (14.96%), and followed by 5′UTR (5.54%) and 3′UTR (3.18%) (Fig. 3E). Consistent with previous studies (Pendleton et al., 2017), a topology study of m6A methylation from our data suggested that the majority of m6A modification sites appeared to be abundant in the protein translation reads regions, such as CDS, stop codon and start codon (Fig. 3F).

**Table 1:** The top 20 differentially methylated peaks.

GeneName	GeneID	Foldchange	Regulation	Chromsome	Peak start	Peak end	Peak_length	*P*_value
Cidea	12683	242.9	Up	chr18	67,343,701	67,343,840	139	0.0000000026518
Cyp2b9	13094	161.748503	Up	chr7	26,210,102	26,210,380	278	0.000000092866
Gprc5b	64297	132.8	Up	chr7	118,995,061	118,995,211	150	0.000000001410
Shh	20423	92.3	Up	chr5	28,457,581	28,458,100	519	0.000000031307
Slc22a26	236149	90.4	Up	chr19	7,781,040	7,781,480	440	0.000000007325
Cyp2b9	13094	89.4313305	Up	chr7	26,201,090	26,201,232	142	0.000000070185
Rgs16	19734	85.3469388	Up	chr1	153,740,348	153,740,460	112	0.000000029918
Ephb2	13844	85.2	Up	chr4	136,654,961	136,655,220	259	0.000000913370
Slc35f2	72022	83.4	Up	chr9	53,816,961	53,817,220	259	0.000001089500
Antxr1	69538	83.4	Up	chr6	87,135,861	87,136,160	299	0.000000028610
Slc1a4	55963	80.8	Up	chr11	20,303,121	20,303,320	199	0.000000057371
Cx3cl1	20312	80.7	Up	chr8	94,781,301	94,781,560	259	0.000000761400
Grin3b	170483	79.4	Up	chr10	79,973,901	79,974,120	219	0.000001848800
Bcl2l14	66813	74.2	Up	chr6	134,432,145	134,432,340	195	0.000004890400
Mical2	320878	68	Up	chr7	112,315,141	112,315,380	239	0.000000888250
Gm5294	384244	67.6	Up	chr5	138,820,181	138,820,186	5	0.000000888230
Pfkfb4	270198	65.3	Up	chr9	109,031,661	109,031,860	199	0.000005620900
Lrrc18	67580	63.8	Up	chr14	33,012,661	33,012,797	136	0.000009528800
Zscan18	232875	62	Up	chr7	12,774,381	12,774,780	399	0.000009404000
Themis	210757	34.4934211	Up	chr10	28,761,281	28,761,620	339	0.000000000000
Tnnt3	21957	376.088889	Down	chr7	142,511,256	142,511,373	117	0.000000042890
Myh1	17879	261.064846	Down	chr11	67,214,941	67,215,307	366	0.000000040426
Tcf15	21407	211.3	Down	chr2	152,148,761	152,149,097	336	0.000000002345
Tcap	21393	210.543103	Down	chr11	98,384,561	98,384,940	379	0.000000028146
Cspg5	29873	208.8	Down	chr9	110,246,821	110,247,160	339	0.000000001822
Mb	17189	195.738806	Down	chr15	77,017,575	77,017,798	223	0.000000031764
Myoc	17926	191.1	Down	chr1	162,648,661	162,649,180	519	0.000000002870
Ttn	22138	174.5	Down	chr2	76,826,074	76,826,152	78	0.000000003882
Trim63	433766	157.5	Down	chr4	134,327,704	134,327,782	78	0.000000003126
Klhl33	546611	140.9	Down	chr14	50,891,561	50,891,941	380	0.000000001160
Klhl38	268807	134.6	Down	chr15	58,314,572	58,314,780	208	0.000000002856
Ankrd23	78321	127.24	Down	chr1	36535,681	36,535,729	48	0.000000010976
Hsd3b5	15496	123.951557	Down	chr3	98,619,301	98,619,520	219	0.000000154970
Kcna5	16493	124.6	Down	chr6	126,533,441	126,533,880	439	0.000000003911
Cspg5	29873	114.5	Down	chr9	110,246,064	110,246,480	416	0.000000000023
Ccl11	20292	105.3	Down	chr11	82,061,678	82,061,790	112	0.000000013596
Lmod3	320502	102.1	Down	chr6	97,247,521	97,247,800	279	0.000000020285
Kiss1	280287	95.1	Down	chr1	133,329,841	133,329,901	60	0.000000089221
Arl2bp	107566	92.2	Down	chr8	94,674,241	94,674,457	216	0.000000002080
Myh2	17882	92.0215827	Down	chr11	67,193,610	67,193,760	150	0.000000017479

### Conjoint analysis of RNA-seq and MeRIP-seq data

**Figure 4:**
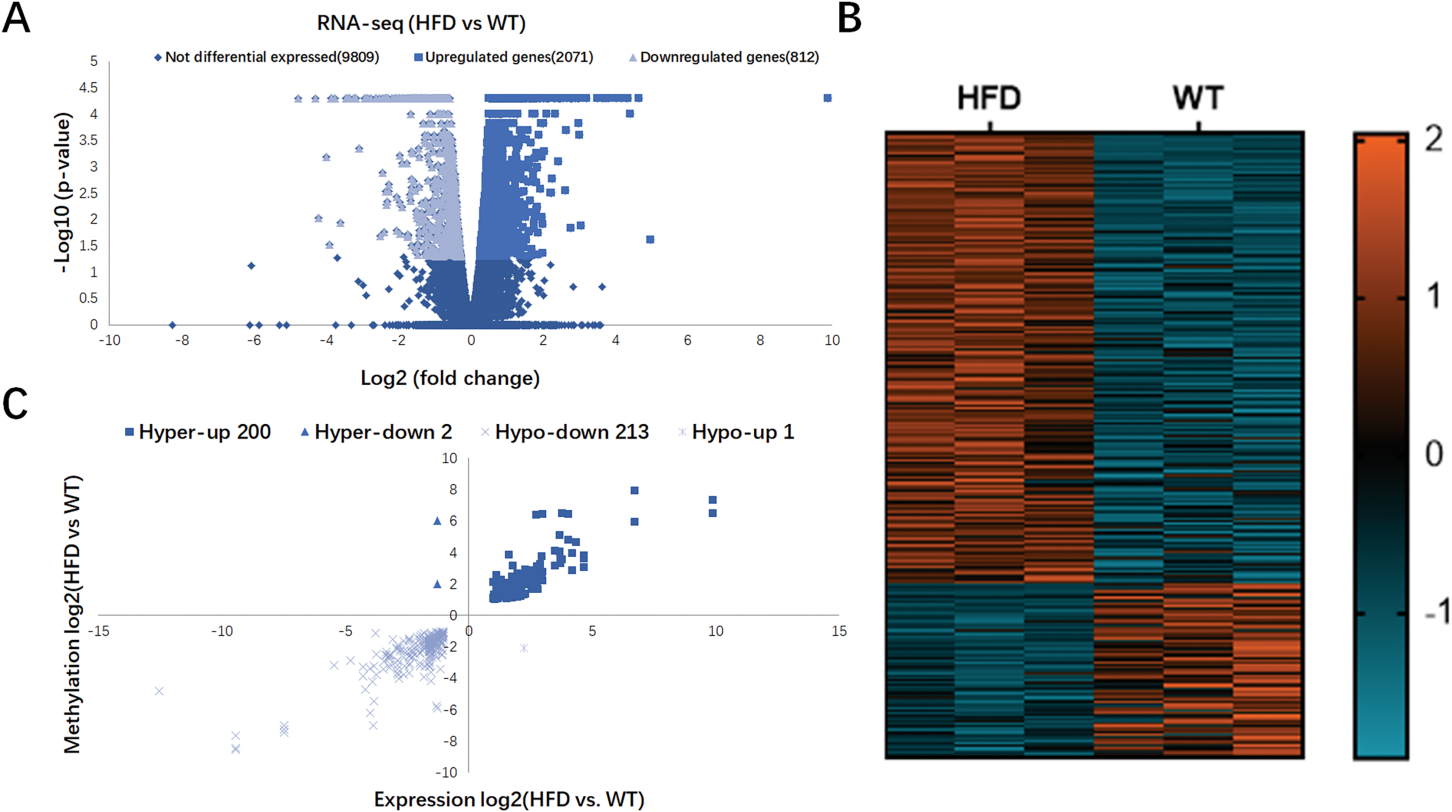
Conjoint analysis of RNA-seq and MeRIP-seq data. (A) Volcano plots exhibiting the differentially expressed genes in HFD and WT mice livers. (B) Heatmap plots presenting the differentially expressed genes in HFD and WT mice livers. (C) Four quadrant plots showing the distribution of differentially expressed genes and differentially methylated genes in HFD and WT mice livers.

In Fig. 4A, RNA-seq data showed 2,883 DEGs in HFD mice compared with the control (fold change ≥ 2 and *P* < 0.05), including 2,071 upregulated genes and 812 downregulated genes. Hierarchical cluster analysis further revealed the distinguished gene expression patterns between two groups (Fig. 4B). By conjoint analysis of RNA-seq and MeRIP-seq data (Fig. 4C), 202 hypermethylated m6A peaks were identified in mRNA transcripts containing 200 upregulated expressed genes (200; hyper-up) and two downregulated expressed genes (2; hyper-down). A total of 214 hypomethylated m6A peaks were identified containing one upregulated expressed genes (1; hypo-up) and 213 downregulated expressed genes (213; hypo-down). The top 20 DEGs containing differentially methylated sites are listed in Table 2.

**Table 2:** The top 20 differentially expressed genes containing diferentially methylated peaks.

Gene	Strand	Fold change	Regulation	*P*-value
Cyp2b9	+	946.0023781	Up	0.00005
Cidea	+	106.1484872	Up	0.04265
Cyp2a22	−	25.42419165	Up	0.00005
Thrsp	−	20.48420504	Up	0.00005
Osbpl3	−	18.23754964	Up	0.00005
Ly6d	−	13.2576209	Up	0.00005
Themis	+	12.8992922	Up	0.00005
Mogat1	+	11.37993175	Up	0.00005
Tceal8	−	7.931149622	Up	0.00005
Gdf15	−	7.913412708	Up	0.00005
Lgals1	+	7.8846133	Up	0.00005
Cidec	−	7.852271194	Up	0.00005
Serpina7	−	7.830964457	Up	0.00005
Pcp4l1	−	5.946043427	Up	0.00005
Abcd2	−	5.87077107	Up	0.00005
Krt23	−	5.639119829	Up	0.00005
Cdkn1a	+	5.548277705	Up	0.00005
Bcl6	−	5.467002366	Up	0.00005
Ifi27l2b	−	5.372747358	Up	0.00005
Wfdc2	+	4.984780575	Up	0.00005
Mup7	−	−13.4412928	Down	0.00005
Cyp4a12b	+	−10.90796446	Down	0.00005
Serpina1e	−	−10.66040246	Down	0.00005
Mup12	−	−10.10326718	Down	0.00005
Cyp4a31	+	−9.141304131	Down	0.00005
Mup1	−	−9.020578762	Down	0.00005
Slco1a1	−	−7.40756818	Down	0.00005
Mup15	−	−7.172668723	Down	0.00005
Des	+	−6.118211584	Down	0.00005
Slc25a4	−	−5.743459549	Down	0.00005
Smad9	+	−4.84188898	Down	0.00005
Mup19	−	−4.473921677	Down	0.00005
Ugt2b37	−	−4.311500359	Down	0.00005
Etnppl	+	−4.198546599	Down	0.00005
Sqle	+	−4.173593235	Down	0.00005
Pck1	+	−3.940612803	Down	0.00005
Ugt2b38	−	−3.90115143	Down	0.00005
Mup21	−	−3.732494152	Down	0.00005
Socs2	−	−3.674225061	Down	0.00005

### GO and KEGG pathway analysis of differentially methylated mRNAs

**Figure 5:**
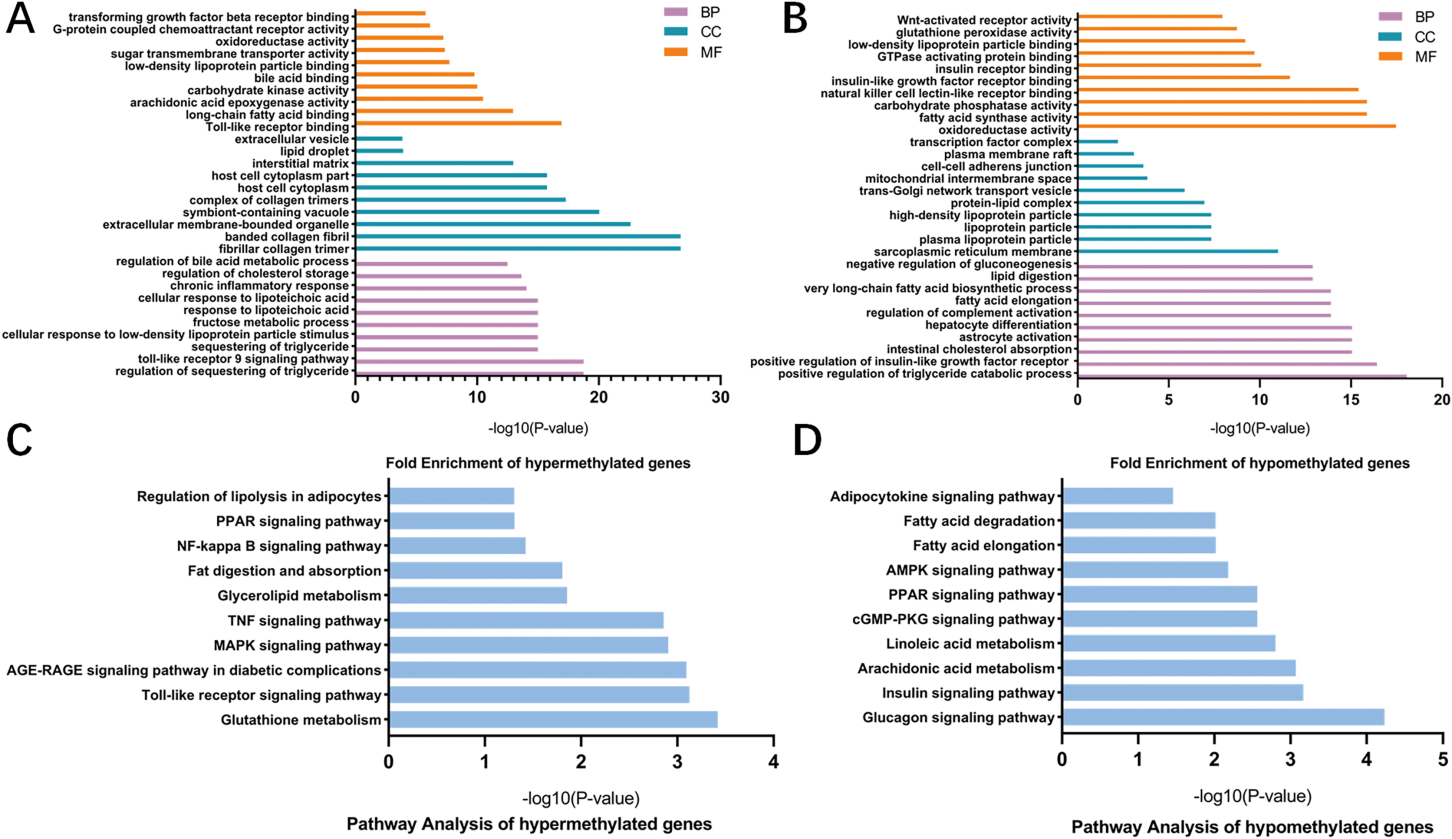
GO and KEGG pathway analysis of differentially methylated genes. (A) GO analysis showing the top 10 GO categories containing hypermethylated genes. (B) GO analysis showing the top 10 GO categories containing hypomethylated genes. (C) KEGG pathway analysis revealing the top 10 pathways of hypermethylated genes. (D) KEGG pathway analysis revealing the top 10 pathways of hypomethylated genes. GO, Gene ontology; KEGG, Encyclopedia of Genes and Genomes; MF, molecular function; CC, cellular components; BP, biological process.

To better understand the roles of m6A modification in NAFLD, GO and KEGG pathway analysis were carried out on the differentially methylated genes. GO analysis revealed that the hypermethylated peaks in HFD mice were involved in the regulation of triglyceride, fructose, bile acid and chronic inflammation (ontology: biological process; Fig. 5A), long-chain fatty acid binding, arachidonic acid epoxygenase activity, oxidoreductase activity and transforming growth factor beta receptor binding (ontology: molecular function; Fig. 5A). The hypomethylated genes were strongly associated with triglyceride catabolic process, lipid digestion and insulin-like growth factor receptor signaling pathway (ontology: biological process; Fig. 5B), fatty acid synthase activity and Wnt-activated receptor activity (ontology: molecular function; Fig. 5B). Notably, KEGG pathway analysis showed that the hypermethylated genes were mainly enriched in fatty acid metabolic pathways, PPAR signaling pathway, Toll-like receptor signaling pathway, MAPK signaling pathway, TNF signaling pathway and NF-κB signaling pathway (Fig. 5C). Additionally, the hypomethylated genes were mainly enriched in insulin signaling pathway, AMPK signaling pathway and cGMP-PKG signaling pathway (Fig. 5D). Collectively, these data indicated the potential mechanisms of m6A methylation in NAFLD progression were closely correlated with the disruption of glucolipid metabolism and activation of hepatic inflammatory responses.

### Both m6A enrichment and expression levels of *CIDEA* were significantly upregulated in HFD mice livers

**Figure 6:**
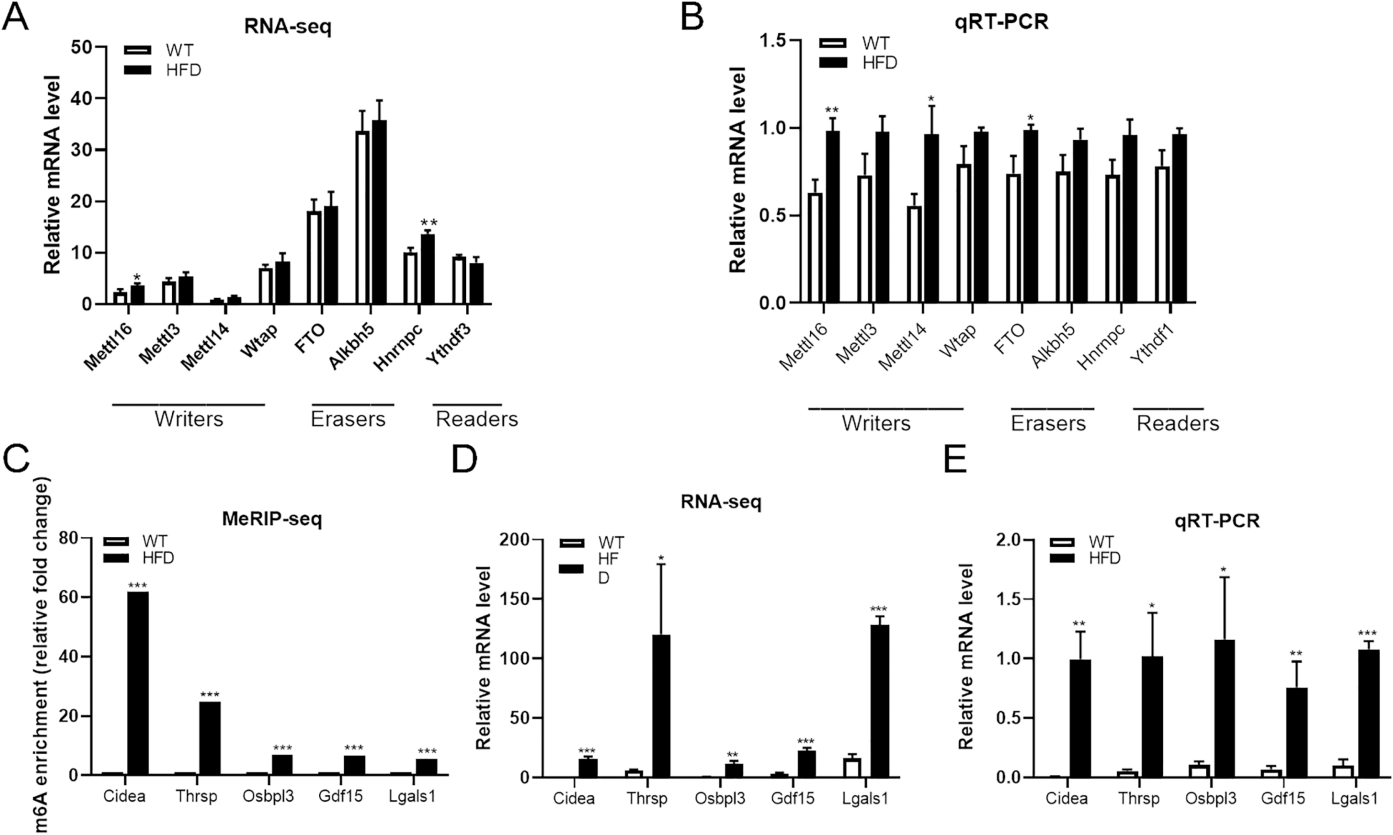
Further analysis of m6A methylation modulators and candidate genes. (A) RNA-seq analysis of m6A methylation modulators including methyltransferase “writers”, demethylases “erasers” and m6A-binding proteins “readers” in HFD and WT mice livers (*n* = 3). (B) The mRNA expression levels of METTL16, METTL14 and FTO were substantially increased in HFD mice livers when evaluated by qRT-PCR (*n* = 3). (C) The m6A enrichment of candidate genes were significantly upregulated in HFD mice livers (*n* = 3). (D) The mRNA abundance of candidate genes were substantially upregulated in HFD mice livers when assessed by RNA-seq (*n* = 3). (E) qRT-PCR analysis of the mRNA expression levels of candidate genes showing that relative mRNA levels were remarkably increased in HFD mice livers (*n* = 3). *P < 0.05; **P < 0.01; ***P < 0.001. The data in (A–E) are presented as the means ± SEM. Candidate genes: *CIDEA*, *THRSP*, *OSBPL3*, *GDF15* and *LGALS1*, selected from the top 20 differentially expressed genes containing differentially methylated sites and have been proven associated with the NAFLD progression.

**Figure 7:**
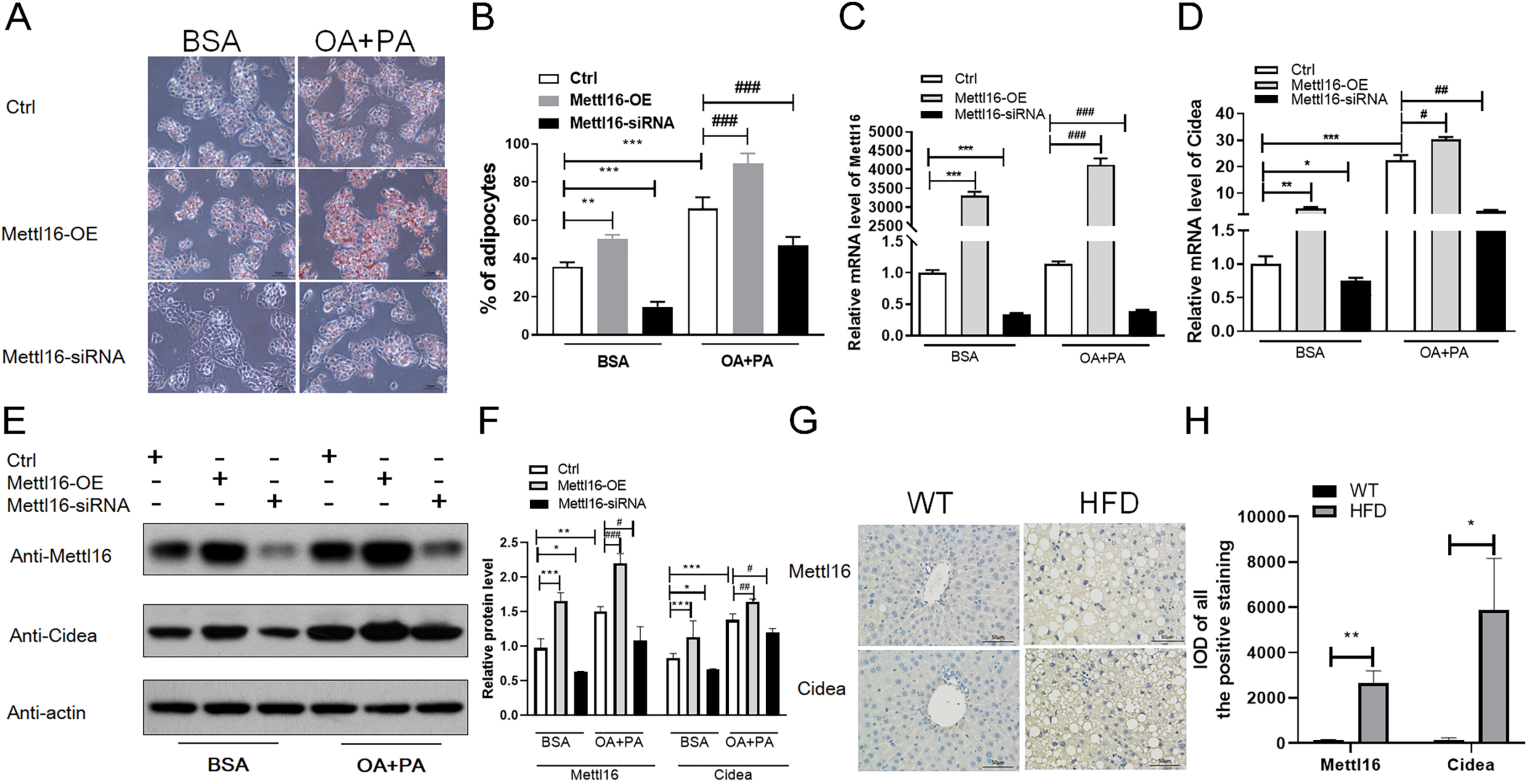
METTL16-mediated m6A methylation promoted the NAFLD progression through upregulating the expression levels of *CIDEA*. (A–F) Following METTL16 overexpression or knockdown in HepG2 cells, oleic-palmitic acid-induced lipid accumulation model was used (*n* = 3); (A) representative microscopic images of HepG2 cells stained with Oil Red O (scale bars, 50 $\mu m$); (B) the percentage of adipocytes in HepG2 cells; (C) qRT-PCR analysis of the mRNA expression levels of METTL16 in HepG2 cells; (D) qRT-PCR analysis of the mRNA expression levels of *CIDEA* in HepG2 cells; (E and F) western blot analysis of the expression levels of *CIDEA* and METTL16 in HepG2 cells. (G) Representative microscopic images of immunohistochemistry analysis of METTL16 and *CIDEA* in mice liver sections (scale bars, 50 $\mu m$). (H) The expression levels of METTL16 and *CIDEA* were significantly upregulated in HFD mice livers when evaluated by immunohistochemistry analysis. *P < 0.05; **P < 0.01; ***P < 0.001; #*P* < 0.05; ##*P* < 0.01; ###*P* < 0.001. The data in (B–D) and (F) (H) are presented as the means ± SEM. BSA, bovine serum albumin; OA+PA; oleic acid+palmitic acid; Ctrl, normal HepG2 cells without METTL16 overexpression or knockdown; METTL16-OE, HepG2 cells were transfected with plasmid to overexpress gene; METTL16- siRNA, HepG2 cells were transfected with METTL16 siRNA to silence gene.

From the top 20 DEGs containing differentially methylated peaks, we selected several genes that have been reported to be involved in NAFLD progression as candidate genes, including *CIDEA*, *THRSP*, *OSBPL3*, *GDF15* and *LGALS1*, for further analysis. MeRIP-seq analysis of these genes revealed that enhanced m6A modifications were found within all of these genes in HFD group, especially *CIDEA* genes (fold change ≥ 240 and *P* = 0.0000, Fig. 6C). RNA-seq analysis of these genes showed that the mRNA abundance of *CIDEA* (*P* = 0.0003), *GDF15* (*P* = 0.0003) and *LGALS1* (*P* = 0.00002) in HFD mice was remarkably elevated after HFD feeding, so as *OSBPL3* (*P* = 0.002) and *THRSP* (*P* = 0.028) (Fig. 6D). Given that *CIDEA* was the the most methylated candidate gene, it was selected as an important target genes for the association between m6A methylation and NAFLD progression in HFD mice. qRT-PCR analysis revealed that *CIDEA* gene was overexpressed at transcriptional level in HFD mice livers (fold change ≥ 1 and *P* = 0.002, Fig. 6E). Next, immunohistochemistry analysis was performed to further validate the expression levels of *CIDEA*. Importantly, the positive particles in liver sections were more diffusely distributed in HFD mice than in WT mice (*P* = 0.012, Figs. 7G and 7H). All together, these outcomes demonstrated that changes in m6A might contribute to the dysregulated gene expressions in NAFLD, especially *CIDEA* gene.

### The expression levels of METTL16 were substantially increased in HFD mice livers

To further explore the driving forces behind differential m6A methylation patterns between HFD and WT mice, we evaluated the mRNA expression levels of major m6A regulators, including writers (METTL16, METTL3, METTL14, WTAP), erasers (FTO, ALKBH5) and readers (YTHDF3, HNRNPC). RNA-seq analysis revealed that the mRNA abundance of METTL16 (*P* = 0.028) and HNRNPC (*P* = 0.005) were substantially elevated in HFD mice livers (Fig. 6A). qRT-PCR analysis showed that the mRNA expression levels of methyltransferases METTL16 were dramatically increased in response to HFD challenge (*P* = 0.004), while other methyltransferases were less remarkably altered (such as METTL14, Fig. 6B). Furthermore, small differences in gene expression were found in the demethylase FTO between two groups (*P* = 0.015, Fig. 6B). By conjoint analysis of RNA-seq and qRT-PCR data, and considering that NAFLD-related genes exhibited enhanced m6A modifications in above analysis, as well as the catalytic function of m6A-related enzymes, METTL16 was selected as a significant marker of the upregulated m6A methylation in HFD mice liver. Immunohistochemistry analysis of METTL16 displayed that positive particles were diffusely distributed in the cytoplasm of hepatocytes in HFD mice, further confirming that the expression levels of METTL16 were significantly elevated in the development of hepatic steatosis (Figs. 7G and 7H).

### METTL16-mediated m6A methylation upregulated the expression levels of *CIDEA* in HepG2 cells

According to previous study (Chung et al., 2019), HepG2 cells treated with oleic and palmitic acid were regularly used to establish NAFLD models *in vitro* by simulating hepatic lipid deposition *in vivo*. To verify whether METTL16-mediated m6A modification indispensably contributed to the overexpression of *CIDEA* in NAFLD, METTL16 transfection and gene silencing with METTL16 siRNA were performed in HepG2 cells to construct the overexpression and konckdown models of METTL16. First, we assessed the expression levels of METTL16 by qRT-PCR analysis, and observed that its mRNA levels were remarkably increased in hepatocytes with METTL16-OE and decreased in that with METTL16-siRNA (Fig. 7C). The western blot analysis further verified that METTL16-OE and Mettl16-siRNA models were successfully constructed as expected (Figs. 7E and 7F). In addition, both the mRNA and protein levels of METTL16 were dramatically increased in hepatocytes treated with FFAs compared with those treated with BSA only (Figs. 7C, 7E and 7F), which was consistent with the outcomes in HFD-induced mice model. Subsequently, qRT-PCR and western blot analysis of *CIDEA* revealed that FFAs led to a significant increase in the mRNA and protein expression levels of *CIDEA* (Figs. 7D–7F) and promoted lipid droplets accumulation (Figs. 7A and 7B) in hepatocytes. Moreover, we observed that forced expression and knockdown of METTL16 dramatically increased and bluntd the expression levels of *CIDEA*, respectively (Figs. 7D–7F). We also observed that FFAs induced higher expression levels of *CIDEA* in METTL16-OE-transfected hepG2 cells compared with controls (Figs. 7D–7F). Taken together, these results revealed that METTL16-mediated m6A methylation would upregulate the expression levels of downstream target genes *CIDEA* in HepG2 cells.

## Discussion

In this study, we explored the significant changes of m6A methylation patterns in HFD-induced mice livers, and found that the mRNA expression levels of NAFLD-associated genes were also simultaneously altered. Intriguingly, we first detected a significant increase in the m6A methyltransferase METTL16 in mice and cell NAFLD models, and further confirmed that the expression levels of downstream target genes *CIDEA* were remarkably upregulated, which implied that METTL16 could become a novel molecular indicator and a potential therapeutic target for NAFLD.

Conjoint analysis of RNA-seq and MeRIP-seq data revealed that elevated m6A methylation levels on *CIDEA*, *THRSP*, *OSBPL3*, *GDF15* and *LGALS1* in HFD mice livers were significantly positively correlated with the mRNA expression levels of these mRNAs (Figs. 6C and 6D). Selected from these lipogenesis genes, *THRSP*, whose mRNA was hypermethylated with 24-fold change and upregulated with over 20-fold change in HFD mice livers, has been confirmed that it would result in lipid accumulation and glucose homeostasis imbalance in human hepatocytes (Zeng et al., 2018). *OSBPL3*, whose mRNA was hypermethylated with 18-fold change and upregulated with seven-fold change in HFD mice livers, has been reported to be closely associated with the progress of hepatic inflammation in advanced stages of NASH (Stein et al., 2017). Additionally, the mRNA level of *GDF15* was hypermethylated with 6.7-fold change and upregulated with 7.9-fold change in HFD mice livers compared with the control. However, *GDF15* has been identified as a protective factor that alleviates fatty acid metabolic dysfunction through enhancing fatty acids oxidation (Zhang et al., 2020b; Zhang et al., 2018). The plausible explanation for the paradoxical results is that biological functions controlled by the target genes are variable in different stages of pathogenesis. The previous research demonstrated that *CIDEA* was strongly associated with the severity of human hepatic steatosis through regulating AMP-activated protein kinase stability (Chang et al., 2006). Sans et al. (2019) reported that the knockdown of *CIDEA* in obese mice significantly reduced the accumulation of lipid droplets in hepatocytes and thus alleviated HFD-induced hepatic steatosis. In this study, the mRNA of *CIDEA* was upregulated with 106-fold change and hypermethylated with approximately 242-fold change in HFD group. Although other candidate genes were also hypermethylated to different degrees, the changes were less than *CIDEA*. Therefore, the most hypermethylated NAFLD-associated gene *CIDEA* was selected for our subsequent research.

Although the pathophysiology of NAFLD remains incompletely clear, it often develops in the context of enhanced liver lipogenesis and imbalanced lipid metabolism (Bessone, Razori & Roma, 2019). Excessive fat accumulation in hepatocytes can lead to lipotoxicity, which further activates Kupffer cells and contributes to hepatocytes damage and mitochondrial dysfunction. Simultaneously, injured hepatocytes also generate a variety of inflammatory cytokines that further accelerate fat accumulation, creating a complicated regulatory network involved with hepatic inflammation and glucolipid metabolism (Farzanegi et al., 2019). Here, hypermethylated genes were mainly concentrated in various important biological pathways associated with the activation of lipid metabolism, such as the regulation of triglyceride and lipolysis, fat digestion and absorption and PPAR signaling pathway (Figs. 5A and 5C). Moreover, we also found that the potential mechanism behind m6A methylation and NAFLD might be closely related to the activation of inflammatory signaling pathways, including Toll-like receptor signaling pathway, TNF and NF-κB signaling pathway (Fig. 5C). Toll like receptors, pattern recognition receptors that recognizes bacterial and viral components on the surface of inflammatory cells such as macrophages and platelets, is activated in NAFLD and its downstream proinflammatory cytokines such as TNF and IL are increased, thereby not only inducing lipogenic enzymes but also leading to liver damage (Todoric et al., 2020; Carpino et al., 2020). NF-κB, a pro-oncogenic factor in NAFLD-HCC development, is also activated in injured hepatocytes to upregulate transcription of inflammatory cytokines, whose increased expressions further activate the downstream NF-κB signal pathway, forming positive feedback that aggravates inflammation and lipid deposition in liver (Wang et al., 2020). Based on above evidence, it is reasonable to hypothesize that altered m6A methylation plays an important role in NAFLD development by inducing inflammatory process and regulating lipid metabolism.

Modifications on mRNA driven by m6A modulators are dynamic and reversible. Wu et al. (2020) reported that resveratrol could alleviate the HFD-induced lipotoxicity through downregulating the expression of YTHDF3 in mice liver, indicating that epigenomics modification manipulated by overexpression or knockdown of methyltransferases or demethylases plays critical roles in metabolic diseases. In this study,the mRNA levels of methyltransferases (METTL14, METTL16) and demethylases (FTO) were significantly upregulated in HFD mice livers by qRT-PCR analysis (Fig. 6B). Data from HFD-induced NAFLD rodent (Chen et al., 2015) and human NASH patients (Lim et al., 2016) demonstrated that the expression levels of FTO were positively correlated to the extent of fat accumulation in hepatocytes. Yang et al. (2019) observed a significant downregulation of weight and fast glucose in patients with silenced FTO expression, which provided a novel therapeutic strategy for the metabolic syndrome. It has been reported that METTL14 is transactivated through NF-κB pathway and further promoted m6A modification within mRNA in NASH model (Feng et al., 2021). However, the mRNA expression levels of FTO and METTL14 were not significantly changed by RNA-seq analysis. Therefore, the correlation of FTO and METTL14 with m6A methylation needs to be further verified in further studies. METTL16, a novel recognized m6A methyltransferase, has been recently identified as a critical regulator involved in various cancer (Wang et al., 2021; Wang et al., 2020; Liu et al., 2019). For instance, the knockdown of METTL16 predicted worse survival and clinical characteristics in HCC patients due to the activation of multiple metabolic pathways (Wang et al., 2020). However, the underlying roles of METTL16 in NAFLD progression haven’t been reported before. In this study, conjoint analysis of RNA-seq and qRT-PCR data revealed that the expression levels of METTL16 were remarkably increased in HFD-induced mice NAFLD model, and many transcript copies of particular genes modified by m6A were changed, especially *CIDEA*. Thus, we hypothesized that the upregulated gene expression of *CIDEA* might be partly attributed to METTL16-mediated m6A methylation in NAFLD model. Subsequently, we further confirmed this assumption by constructing overexpresion and knockdown of METTL16 model in HepG2 cells. As a result, we observed a significant increase in the expression levels of downstream target genes *CIDEA* in HepG2 cells with METTL16 overexpression.

There are certain limitations to this study as well. Gender differences would lead to different disease features. Therefore, single male mouse study may overlook the impact of sex differences on the disease spectrum. Furthermore, the content of lipids and their metabolites during the steatosis of liver were not investigated in this research. Therefore, it is worthwhile to perform the lipid content measurements and lipase assays in further work to clarify how m6A methylation affects specific components of lipid metabolism.

In summary, we uncovered the distinguished m6A modification mediated by METTL16 was engaged in post-transcriptional regulation of *CIDEA* in mice and cell models of NAFLD. These results enhance our understanding of METTL16-associated m6A methylation regulatory network in the pathogenesis of NAFLD.

## Conclusions

Our study illustrated the m6A methylation profiles in HFD-induced mice NAFLD model,and we identified for the first time that METTL16 would upregulate m6A methylated level of *CIDEA* mRNA. These results imply that METTL16 could become a novel molecular indicator and potential therapeutic target for NAFLD in the future research.

## Supplemental Information

10.7717/peerj.14379/supp-1 Supplemental Information 1 Primers for reverse transcription quantitative polymerase chain reaction.

10.7717/peerj.14379/supp-2 Supplemental Information 2 Raw data.

10.7717/peerj.14379/supp-3 Supplemental Information 3 Author Checklist-Full.

## Additional Information and Declarations

### DNA Deposition

The following information was supplied regarding the deposition of DNA sequences: The raw data is available at NCBI GEO: GSE202843, in: https://www.ncbi.nlm.nih.gov/geo/query/acc.cgi?acc=GSE202843. The following secure token has been created to allow review of record GSE202843: kdihieeqrpylzsx.

### Data Availability

The following information was supplied regarding data availability: The following information was supplied regarding data availability: The raw data is available at NCBI GEO, accession numbers: GSE202843, in: https://www.ncbi.nlm.nih.gov/geo/query/acc.cgi?acc=GSE202843. PCR raw data are provided as Supplemental Files.
