# Supplementary material for: METTL16-mediated translation of CIDEA promotes non-alcoholic fatty liver disease progression via m6A-dependent manner
Source: PeerJ. 2022 Dec 1;10:e14379. doi: 10.7717/peerj.14379 (PMC9744165; doi:10.7717/peerj.14379)
Supplement: Supplemental Information 1 [file peerj-10-14379-s001.docx]

| Supplemental table 1 Primers for reverse transcription‑quantitative polymerase chain reaction. | | |
| --- | --- | --- |
| Gene | Forward primer, 5'‑3' | Reverse primer, 5'‑3' |
| Mettl16 | GCAGATCATCGCAGCCAAAG | ACTGTTGCCCTTGTCACCAA |
| Mettl3 | GCACTTCAGACGGATTATCAAC | TCATAGTGGACATACTTGCAGG |
| Mettl14 | GCCGACAGATTTGAAGAATACC | CACATCAAACTTGGGTGTCAAT |
| Wtap | TGAACTGAGTGCCTGGAAGT | CTTTCTCGAGGTGGGACTGT |
| FTO | TACGTGGCATGTTGGTTTTAAG | CATCCAGCATGAAATAGCAGTC |
| Alkbh5 | CTGTATGTGACATGCTTACACG | TCTGTGGGCAACAACTAACTAT |
| Hnrnpc | AAATTGTGGGCTGCTCTGTG | GGTTCACTTTTGGCTCTGCA |
| Ythdf1 | GGGCAGACAGGTTTTCACAG | ATCCCCAATCTTCAGGCCAA |
| Cidea | TGGTGGACACAGAGGAGTTC | GTGACTCTGGCTATTCCCGA |
| Thrsp | ACCTAGAAGCCCAGTTCCAC | GAACCTGCCCTGTCATTTCC |
| Gdf15 | CGGTGGTTCTTATGCACAGG | GAGTGTAGGTGAGGAGCAGG |
| Osbpl3 | ATGACTTACACCCAGGAGCC | AGAATCTCTTGTGCCAGCCT |
| Lgals1 | CTCTCTTGTGCTTTCCAGGC | TTCATCCATCCACCTCCCAC |
| GAPDH | AAGGTCATCCCAGAGCTGAA | CTGCTTCACCACCTTCTTGA |
